# Idiopathic Scrotal Calcinosis: A Case Report and Review of Postoperative Outcomes

**DOI:** 10.1155/2020/8877695

**Published:** 2020-09-14

**Authors:** Mathew Yamoah Kyei, Robert Djagbletey, Afua Darkwa Abrahams, James Edward Mensah

**Affiliations:** ^1^Department of Surgery and Urology, University of Ghana Medical School, P.O. Box 4236, Accra, Ghana; ^2^Department of Anaesthesia, University of Ghana Medical School, P.O. Box 4236, Accra, Ghana; ^3^Department of Pathology, University of Ghana Medical School, P.O. Box 4236, Accra, Ghana; ^4^Department of Surgery and Urology, University of Ghana Medical School, P.O. Box 4236, Accra, Ghana

## Abstract

Idiopathic scrotal calcinosis is a rare condition, characterized by the idiopathic deposition of calcium in the scrotal dermis leading to the formation of a single nodule or multiple nodules of different sizes. Surgical excision of the nodules reduces symptoms and improves cosmesis. We present a case of idiopathic scrotal calcinosis that had an en bloc excision of scrotal skin nodules and primary closure of the scrotal skin. Handling each hemiscrotum as a separate entity and preserving the median raphe with its uninvolved skin improved the cosmesis. Reported outcomes of surgery were satisfactory with no postoperative complications. At 30 months of follow-up, the residual scrotal skin had regained its laxity and the scrotum its normal configuration. There is the risk of recurrence of the calcific nodules post excision, but these may be smaller in size and with regained scrotal configuration that could be amenable to excision with further preservation of the native scrotal skin.

## 1. Introduction

Idiopathic scrotal calcinosis is a rare condition characterized by the idiopathic deposition of calcium in the scrotal dermis leading to the formation of a single nodule or multiple nodules of different sizes. Historically, it was first reported by Lewinski in 1883 and named as idiopathic scrotal calcinosis by Shapiro in 1970 [1]. It is benign and common in the third decade of life.

Patients usually present because of concern about cosmesis and itching which adversely affect their quality of life. Other reported presentations include pain, superimposed infection, and exudation of chalky substances [2].

There is no underlying metabolic disorder with normal serum calcium, phosphate, and parathyroid hormone levels [3].

Imaging studies such as CT scans will show calcifications in the scrotal wall [4] but are usually not needed as diagnosis is mainly clinical and confirmed with histology of excised nodules.

Surgery is the treatment approach, and it is to reduce symptoms and improve cosmesis [5]. This involves en bloc excision of the scrotal wall containing the nodules with/or selected excision of each nodule [2]. The aim is to excise all nodules while leaving enough scrotal skin to allow for primary closure or scrotoplasty using the native scrotal skin.

We present a case of idiopathic scrotal calcinosis that had an en bloc excision of scrotal skin nodules and primary closure of the scrotal skin. Handling each hemiscrotum as a separate entity and preserving the median raphe with its uninvolved skin improved the cosmesis. The residual scrotal skin regained its laxity with the scrotum assuming its normal configuration at 30 months of follow-up. The patient gave consent for publication of this case report.

We also reviewed the postoperative outcomes after surgical intervention for idiopathic scrotal calcinosis.

## 2. Case Presentation

A 32-year-old married man presented in December 2017 with slow-growing multiple nodules on the scrotum of 5-year duration.

He self-reported because of cosmesis and occasional mild itch. He indicated he had been applying talcum powder to the scrotum for many years aiming to reduce sweating in the area. There was no history of trauma or infection of the scrotum. He also had no systemic or metabolic disease.

**Figure 1:**
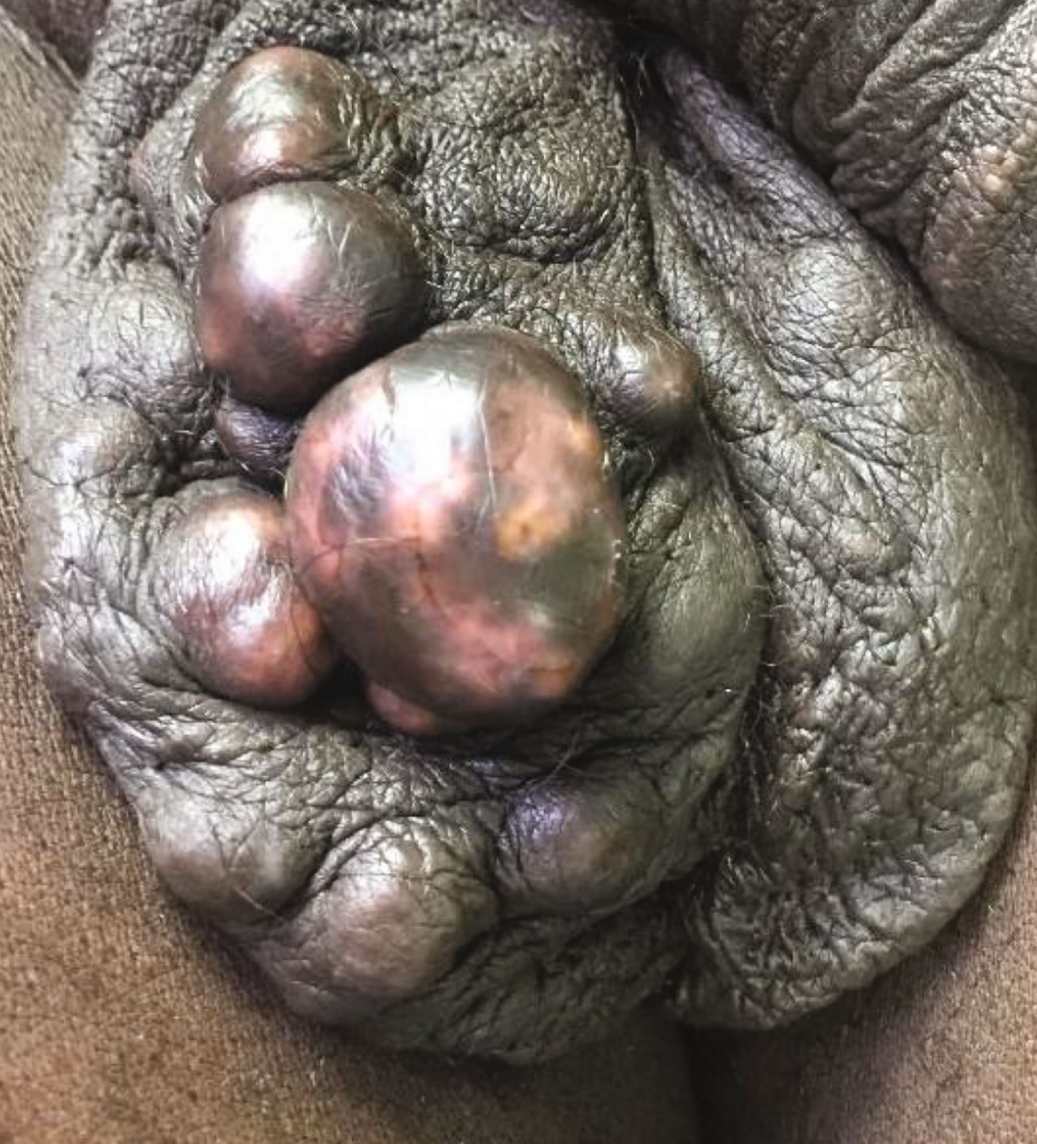
Showing the scrotum with multiple intradermal nodules of different sizes.

On examination, the scrotum had multiple, nontender, hypopigmented, firm nodules of different sizes that were intradermal. The largest was about 25 mm in size. It was bilateral but much larger nodules on the right hemiscrotum. The testes were normal, and there was no inguinal lymphadenopathy (Figure 1).

His serum calcium, phosphate, and parathyroid hormone levels were normal.

An important differential considered was multiple epidermal cysts of the scrotum.

The patient consented for an excision biopsy of the scrotal nodules.

The procedure was done under spinal anaesthesia using 2.5 mls of 0.5% hyperbaric bupivacaine through the L4-L5 interspace with a gauge 26 Whitacre needle.

The patient was positioned supine, and the site was cleaned with povidone-iodine and draped. The scrotum was inspected, and all nodules were noted considering each hemiscrotum as a unit. On careful inspection, the skin over the median raphe was noted not having any nodules. A decision was made to preserve it.

A wide local excision of the involved scrotal skin was made removing the nodules en block while taking each hemiscrotum as a unit. This required a semielliptical incision on the lateral aspects and a vertical paramedian raphe incision on the medial aspects. Dissecting was just below the dermis so as not to disrupt the dartoic muscle layer which was spared.

**Figure 2:**
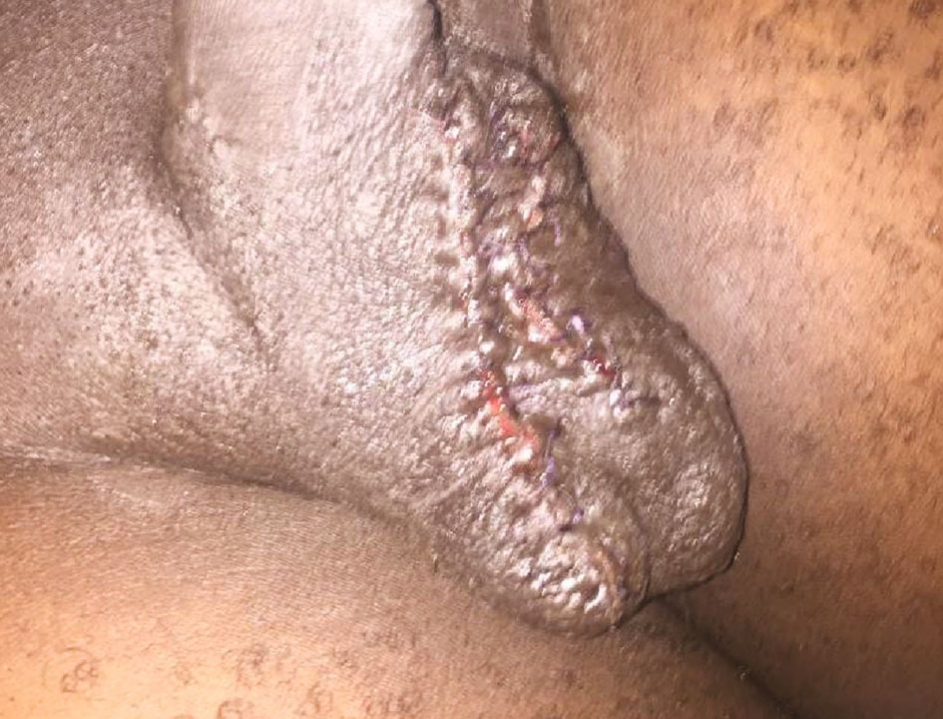
Scrotum four days post scrotoplasty with preserved skin over the median raphe.

The wound was then closed with vicryl 2-0 using an interrupted technique (Figure 2).

**Figure 3:**
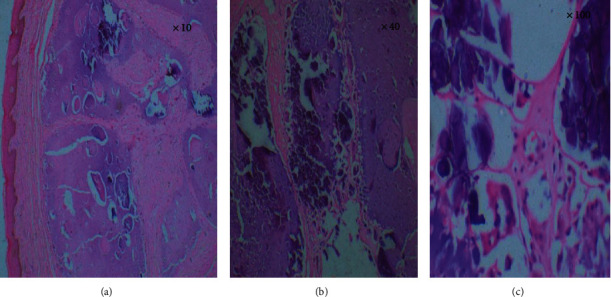
Histology of excised tissue showing (a) intact overlying epidermis with calcium deposits in the dermis (×10), (b) calcium deposits in the dermis (×40), and (c) inflammatory reaction around the deposits (×100).

The histology of the excised specimen showed dense fibrosis of the dermis with foci of calcifications surrounded by multinucleated giant cells of the foreign body type. The overlying epidermis was normal confirming the diagnosis of scrotal calcinosis (Figures 3(a)–3(c)).

**Figure 4:**
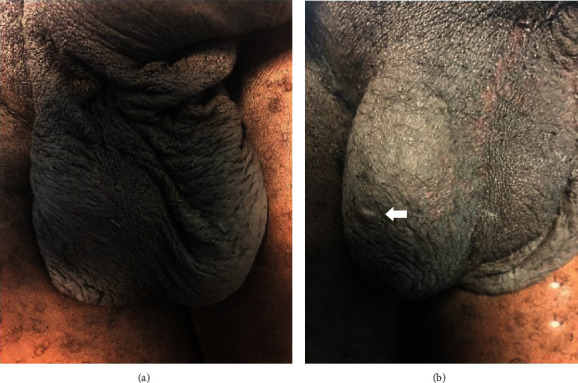
(a) Scrotum having regained the normal configuration. (b) A 5 mm recurrent calcific nodule (arrowed) on the right hemiscrotum at 30 months of follow-up.

Thirty months post excision at follow-up, the natural laxity and configuration of the scrotum had returned and the patient was satisfied with the outcome. A recurrence of a single nodule (5 mm) on the right hemiscrotum was however observed, but it was of no bother to the patient and he therefore declined excision (Figures 4(a) and 4(b)).

## 3. Discussion

Idiopathic scrotal calcinosis is a rare condition with debated aetiology. While no underlying aetiology has been widely accepted, some researchers have indicated that the cause could be attributed to dystrophic deposition of calcium in an existing epidermal cyst, eccrine epithelial cyst, or degenerated dartoic muscle [5, 6]. Most patients present for cosmetic reasons as well as itching as seen in this patient [2]. The patient at 32 years had had the initial nodules when he was 27 years old supporting the observation that it tends to occur in the third decade.

The examination findings of multiple firm, intradermal, nontender nodules of different sizes and brownish in colour (hypopigmented in a dark skin) are the typical examination findings [2]. There is usually no associated inguinal lymphadenopathy as was observed in this case supporting the benign nature of the condition.

The absence of an underlying abnormality in calcium metabolism in idiopathic scrotal calcinosis [3] was also observed as the serum calcium, phosphate, and parathyroid hormones of the patient were normal.

Surgical excision for histology, limited to the involved scrotal skin but ensuring removal of all nodules, confirms the diagnosis and also treats the condition. The surgery so offered also provides the needed cosmesis. The procedure is usually performed under local or spinal anaesthesia [5, 7]. In this current case, the procedure was done under spinal anaesthesia. The use of spinal anaesthesia allows time for the performance of more complex reconstruction that may take a longer duration to accomplish. Careful inspection of the scrotum taking into consideration the number of nodules present and the extent of involvement of the scrotum allows one to plan the excision process. This could be excising each nodule separately using elliptical incisions or en bloc as a wide local excision or combination of both techniques [2, 5, 7]. The aim is to preserve adequate scrotal skin for the reconstruction and so maintain the scrotal function.

While the median raphe is preserved if the nodules involved only one hemiscrotum, it tended to be part of the en bloc excision when both ventral hemiscrota are involved [5, 8]. In this case, we performed a wide local excision of the scrotal skin with the involved nodules en bloc considering each hemiscrotum as a unit. This allowed for the preservation of the skin over the median raphe. The decision to preserve the median raphe with its overlying skin was done because it was not involved and the incision used was able to allow for the complete removal of the nodules. An involved median raphe skin if observed needs to be excised to allow for complete removal of the nodules so as to prevent recurrence. Hedhli et al. recently published their report where they excised the lesion on either side of the median raphe and centered their scrotal reconstruction on the scrotal median raphe to provide a scrotal lift shape [9].

The suture used in this case was vicryl 2-0 (absorbable suture) which worked well with no complications. Other reports have preferentially used nonabsorbable sutures such as 3-0 prolene for the reconstruction [7].

This presentation showed that the preservation of the median raphe of the scrotum with its skin, if not involved, enhances the outcome in terms of cosmesis. There was also the regain of the scrotal laxity and configuration over time.

The finding in this case as reported by others is that of calcium deposition in the dermis with associated granulomatous reaction [9]. There was a giant cell reaction in this case reported as observed in other reports [5].

Though there is a debate about the probability of recurrence of the calcific nodules after excision, this case demonstrated a single recurrence at two and half years (30 months) of follow-up supporting views of risk of recurrence after excision [10]. However, the recurred nodule with its size (5 mm) in relation to the regained laxity and configuration of the scrotum could allow for a second excision if so desired with further preservation of the scrotal skin and cosmesis.

Most authors reported no postoperative complications and satisfactory or good cosmetic outcomes after surgical excision and primary closure.

Pompeo et al. (multiple nodules) reported no recurrence at 3 months of follow-up [2]. Both Khallouk et al. (multiple nodules) and Tsai et al. (single nodule) reported no recurrence at 12 months of follow-up [5, 12]. A single recurrent nodule of size 5 mm was seen in our case at 30 months of follow-up.

## 4. Conclusion

Surgery is the mainstay of treatment of idiopathic scrotal calcinosis with an overall good cosmetic outcome. Preserving the median raphe with its uninvolved skin improved the aesthetics after surgery. The residual scrotal skin regained its laxity with the scrotum assuming its normal configuration at follow-up. There is the risk of recurrence of the calcific nodules, but these may be small in relation to the regained configuration of the scrotum and thus could be amenable to excision with further preservation of the native scrotal skin.
